# Anticipation of Uncertain Threat Modulates Subsequent Affective Responses and Covariation Bias

**DOI:** 10.3389/fpsyg.2018.02547

**Published:** 2018-12-11

**Authors:** Zhiling Qiao, Haiyang Geng, Yi Wang, Xuebing Li

**Affiliations:** ^1^Key Laboratory of Mental Health, Institute of Psychology, Chinese Academy of Sciences, Beijing, China; ^2^Department of Psychology, University of Chinese Academy of Sciences, Beijing, China; ^3^Shenzhen Key Laboratory of Affective and Social Cognitive Science, Shenzhen University, Shenzhen, China; ^4^Center for Emotion and Brain, Shenzhen Institute of Neuroscience, Shenzhen, China; ^5^BCN Neuroimaging Center, University Medical Center Groningen, University of Groningen, Groningen, Netherlands

**Keywords:** uncertainty, altered anticipation, covariation bias, affection, ERP

## Abstract

Uncertainty contributes to stress and anxiety-like behaviors by impairing the ability of participants to objectively estimate threat. Our study used the cue-picture paradigm in conjunction with the event-related potential (ERP) technique to explore the temporal dynamics of anticipation for and response to uncertain threat in healthy individuals. This task used two types of cue. While ‘certain’ cues precisely forecasted the valence of the subsequent pictures (negative or neutral), the valence of pictures following ‘uncertain’ cues was not predictable. ERP data showed that, during anticipation, uncertain cues elicited similar Stimulus-Preceding Negativity (SPN) to certain-negative cues, while both of them elicited larger SPN than certain-neutral cues. During affective processing, uncertainty enlarged the mean amplitude of late positive potential (LPP) for both negative and neutral pictures. Behavioral data showed that participants reported more negative mood ratings of uncertain-neutral pictures relative to certain-neutral pictures and overestimated the probability of negative pictures following uncertain cues. Importantly, the enlarged anticipatory activity evoked by uncertain cues relative to that evoked by certain-neutral cues positively modulated the more negative mood ratings of uncertain-neutral pictures relative to certain-neutral pictures. Further, this more negative mood ratings and the general arousal anticipation during anticipatory stage contributed to the covariation bias. These results can provide a novel insight into understanding the neural mechanism and pathological basis of anxiety.

## Introduction

Uncertainty, the unknown probability, time and cost of a future event, is a phenomenon that we encounter in our everyday lives. When the occurrence of an event is uncertain, identifying its relationship with the current circumstance has survival benefits, which enables people to do mental and motivational preparations and achieve desired outcomes (Alloy and Tabachnik, 1984). However, people show inability to objectively assess the relationship between environmental cues and potential outcomes and tend to highly estimate the frequency of threat (Grupe and Nitschke, 2011). Uncertainty also enlarges the processing of stimuli and leads to more negative experience of stimuli (Grupe and Nitschke, 2011; Dieterich et al., 2016). Grupe and Nitschke (2013) proposes the model ‘Uncertainty and Anticipation Model of Anxiety’ and highlights the core role of anticipation in the processing of uncertainty. The model suggests that, rather than exaggerated responses elicited by uncertain threat, it is the anticipation of this threat that is the key to understanding the neuropathological basis of clinical and subclinical anxiety disorders. However, we still know little about the neural mechanisms of anticipation of future threat when its occurrence is uncertain. Whether the neural anticipatory activity is altered by uncertainty? Whether uncertain anticipation modulates participants’ subsequent neural and behavioral responses to stimuli? What are the contributors to people’s inability to objectively assess the relationship between uncertain situation and threat?

When the occurrence of a negative event is uncertain, individuals’ common and seemingly automatic reflection is to estimate the probability of the event. Using the cue-picture paradigm, Grupe and Nitschke (2011) detected the expectancy bias under uncertainty. That was participants tended to anticipate negative pictures after uncertain cues. In this task, participants were required to passively view either negative or neutral pictures which were preceded by three types of cue (certain-negative cue, certain-neutral cue or an uncertain cue). While both the certain-negative cue and the certain-neutral cue always predicted the negative and neutral pictures, respectively, the uncertain cue predicted either a negative or neutral picture according to a 50/50 ratio. Participants, however, were given no information about this percentage prior to the experiment, and were asked to report their anticipation of the percentage of negative picture following uncertain cues by rating at the expectancy scale prompted “Expect aversive picture?” The result indicated that individuals thought uncertain cues were more likely to be followed by negative pictures, which was deemed as expectancy bias. This directly reported index suggests that people have altered anticipation for uncertain future threat. However, whether this altered anticipation could be detected at neural level is still unclear.

Stimulus-Preceding Negativity (SPN) is a slow cortical event-related potential (ERP) component and is observed during the anticipation of a forthcoming stimulus (Van Boxtel and Böcker, 2004). Studies have identified that the presentation of pictures varying in emotional content can reliably yield this component (Simons et al., 1979; Klorman and Ryan, 1980; Lumsden et al., 1986; Amrhein and Pauli, 2005), specifically, emotional salience pictures yield larger SPN than neutral pictures (Klorman and Ryan, 1980; Bradley et al., 2001). The enlarged amplitude of SPN is evidenced to be more related to arousal rather than valence. Both high arousal negative and positive stimuli can produce pronounced SPN (Poli et al., 2007) by activating defensive and appetitive motivational systems, respectively (Bradley et al., 2001). Thus, our study would use the cue-picture paradigm with ERP technique to explore the anticipatory activities reflected by the amplitude of SPN. The categories of materials included were neutral and negative pictures, which were the same as those included in previous studies to research uncertain anticipation (Sarinopoulos et al., 2010; Grupe and Nitschke, 2011; Dieterich et al., 2016, 2017). Positive pictures were not recruited, since they might interfere with the detection of biased anticipation for negative pictures by eliciting a pronounced SPN (Poli et al., 2007). In addition, in order to capture participants’ automatic and natural anticipatory arousal, our study would not include the expectancy rating scale. Although the intention of this scale is to explicitly detect the anticipatory rating of participants, it may also have the unintended consequences of contaminating the natural neural anticipatory activities. For example, the expectancy scale forces participants to make an explicit choice about the outcome and thus enter a different state of anticipation compared to the more natural state in which the possibility of either a negative or neutral outcome remains unresolved. Based on previous studies (Bradley et al., 2001; Grupe and Nitschke, 2011), we supposed to see larger SPN after certain-negative and uncertain cues relative to certain-neutral cues.

Expectancy is of great importance since it can dramatically influence people’s responses to a diversity of events. For example, placebo reduces participants’ reported pain of shock (Wager et al., 2004), and misleading the expectancy of aversive taste can dampen participants’ reports of the aversiveness of the taste (Nitschke et al., 2006). Thus, when the occurrence of threat is uncertain, the anticipation of the potential outcome may be an important factor in participants’ perception of and response to the outcomes. During the study of Grupe and Nitschke (2011), participants were asked to rate their mood after viewing each picture. Results showed that participants reported more negative mood ratings of uncertain-negative pictures relative to certain-negative pictures, indicating uncertainty intensified emotional experience. This uncertainty-related effect on emotion has also been evidenced in other study (Bar-Anan et al., 2009), which might be related to the expectancy bias of uncertain threat. Thus, we expected to see more negative mood ratings of pictures under uncertainty compared to certainty and that these more negative mood ratings would be partly attributed to the altered anticipatory activities under uncertainty.

The anticipatory activities have also been evidenced to be associated with the neural processing of subsequent stimuli. Using the cue-picture paradigm in conjunction with functional magnetic resonance imaging (fMRI), Sarinopoulos et al. (2010) found the activation of anterior cingulate cortex (ACC) during the anticipatory stage, a region referring to the anticipation-driven modulatory function and regulation of emotional response (Petrovic et al., 2005; Phan et al., 2005; Etkin et al., 2006; Sarinopoulos et al., 2006; Egner et al., 2008), and the activations of insula and amygdala during processing of pictures, two regions implicated in interoception and vigilance for motivational salience separately (Craig, 2002; Critchley, 2004, 2005). In particular, the anticipatory activities of ACC elicited by uncertain cues relative to certain-negative cues were inversely related to enhanced activations of insula and amygdala responding to uncertain-negative pictures relative to certain-negative pictures. These results suggest a modulation effect of anticipatory activities on the subsequent neural processing of pictures. However, considering the low temporal resolution of fMRI, which might limit the explanation of these results, ERP would be a useful tool to further probe into this question by precisely separating cue stage from picture stage.

Studies of uncertainty using ERP technique showed that pictures under uncertain condition elicited larger amplitudes of P2 and late positive potential (LPP) than pictures under certain condition (Dieterich et al., 2016). P2, a positive deflection at frontal–central areas, beginning around 200 ms after the presentation of stimulus, reflects the early selective attention and is larger for stimuli of emotional salience (Dan and Hajcak, 2008; Carretié et al., 2013). LPP, a slow positive deflection, beginning around 300 ms after the presentation of a stimulus, is enlarged by motivational stimuli (Cuthbert et al., 2000). The enlarged P2 and LPP for uncertain stimuli relative to certain stimuli (Dieterich et al., 2016) indicated that uncertainty increased the attention allocation to the processing of stimuli, which was consistent with the fMRI study (Sarinopoulos et al., 2010). Thus, in our study, we expected to see that uncertainty would enlarge the amplitude of P2 and LPP for both negative and neutral pictures. In addition, based on the study of Sarinopoulos et al. (2010), we assumed that the anticipatory activities reflected by the mean amplitude of SPN would negatively modulate the neural responses to pictures reflected by the mean amplitude of LPP.

During the cue-picture paradigm, participants were also asked to assess the frequency of negative pictures following uncertain cues at the end of the task (Grupe and Nitschke, 2011). Results showed that, though participants had been multiply exposed to the relationship between uncertain cues and potential outcomes, they still misjudged the possibility of uncertain cues followed by negative pictures, which was described as a covariation bias. Covariation bias has been observed in studies using fear-relevant cues (Barlow, 1988; de Jong et al., 1995b; Amin and Lovibond, 1997; Buhr and Dugas, 2002; Hermann et al., 2004), indicating that uncertain cue may play a role akin to the fear-relevant cue. This increased estimation of relationship between uncertainty and threat was evidenced to be partly attributed to the expectancy bias (Grupe and Nitschke, 2011). That was, the more participants expected a negative picture following the uncertain cue, the larger they reported the frequency of negative pictures following uncertain cues at the end of the experiment. This modulated effect was also detected on neural level. Sarinopoulos et al. (2010) found the anticipatory activity of ACC was positively correlated with the post-experimental estimations, suggesting that the anticipatory activities under uncertainty might predict or at least partly account for the covariation bias. So, our study supposed to see the covariation bias reflected by overestimation of the frequency of negative pictures following uncertain cues and the positive correlation between this bias and uncertain anticipatory activities (the amplitude of SPN under uncertainty).

One thing to be noted in the study of Grupe and Nitschke (2011) is that, though participants presented the expectancy bias, they didn’t report covariation bias. This inconsistency of expectancy bias and covariation bias is also reported in previous studies using fear-relevant cues. Expectancy bias of threat after fear-relevant cues was frequently reported in both healthy populations and anxiety patients (Mcnally and Heatherton, 1993; Amin and Lovibond, 1997; Kennedy et al., 1997) while the covariation bias was rarely detected in healthy populations and was consistently observed in patients (de Jong et al., 1995b; Pauli et al., 1998). This inconsistency indicates that covariation bias might be partly attributed to but not fully arise from expectancy bias. Covariation bias is supposed to reflect an affective matching that people tend to display a bias to associate emotional stimuli with the matching emotional contexts (Tomarken et al., 1989, 1995). For example, people overestimate the frequency of shocks following the presentation of fearful stimuli compared to innocuous stimuli (Tomarken et al., 1989, 1995; de Jong et al., 1992). This affective matching is of emotion specificity, that anger and sadness bias the likelihood estimations of angering and sad events, separately (Desteno et al., 2000). The study of Vanoyen and Vrana (2000) used four kinds of materials (negative valence, high arousal; negative valence, low arousal; positive valence, high arousal; positive valence, low arousal) in conjunction with light flash, as a visual startle-producing stimulus, explored how both valence and arousal affected the covariation bias (the overestimation of the frequency of flash light). Results showed that participants reported larger percentage of startle probes occurring during high-arousal imagery than during low-arousal imagery. Also, they reported larger percentage of startle probes occurring during negative imagery than positive imagery. These results suggest that both valence and arousal dimensions of emotion influence the post-experimental estimations. Based on previous study (Grupe and Nitschke, 2011), uncertainty produced more negative mood ratings of pictures. Thus, we assumed the more negative mood ratings of pictures caused by uncertainty might be an important contributor to covariation bias.

In conclusion, our study, using cue-picture paradigm in conjunction with ERP technique, would explore the temporal dynamics of stimuli processing under uncertainty and the modulation effect of anticipation on subsequent responses to stimuli and covariation bias. First, we hypothesized that certain-negative cues and uncertain cues would elicit larger SPN compared to certain-neutral cues during the cue stage and negative pictures would elicit larger P2 and LPP relative to neutral pictures and uncertainty would enlarge the amplitude of P2 and LPP during picture stage. For behavioral reports, more negative mood ratings were expected for negative pictures relative to neutral pictures and for pictures under uncertainty compared to certainty. Second, we hypothesized that altered anticipatory activities by uncertainty would contribute to more negative mood ratings of pictures. Specifically, anticipatory activities elicited by uncertain cues relative to certain-negative cues (the difference mean amplitude of SPN) would contribute to more negative mood ratings of uncertain-negative pictures relative to certain-negative pictures. Similarly, anticipatory activities elicited by uncertain cues relative to certain-neutral cues (the difference mean amplitude of SPN) would contribute to more negative mood ratings of uncertain-neutral pictures relative to certain-neutral pictures. Third, we hypothesized that the neural anticipatory activities would modulate the subsequent neural processing of pictures. Specifically, anticipatory activities elicited by uncertain cues relative to certain-negative cues (the difference mean amplitude of SPN) would be inversely related to neural processing of uncertain-negative pictures relative to certain-negative pictures (the difference mean amplitude of P2 and LPP). Similarly, anticipatory activities elicited by uncertain cues relative to certain-neutral cues (the difference mean amplitude of SPN) would be inversely related to neural processing of uncertain-neutral pictures relative to certain-neutral pictures (the difference mean amplitude of P2 and LPP). Fourth, there would be a covariation bias that participants would overestimate the percentage of negative pictures following uncertain cues and this bias would be attributed to both the anticipation of uncertain threat (the amplitude of SPN elicited by uncertain cue) and the more negative mood ratings of pictures under uncertain condition relative to certain condition.

## Materials and Methods

### Participants

Thirty-one healthy individuals (seventeen males; mean age ±*SD* = 22.8 ± 1.76) made up of undergraduate and graduate students were recruited from colleges in Beijing, China to participate in our study. All were right handed, had normal or corrected to normal vision and reported no current or past history of neurological or psychiatric disorders. Written informed consent was obtained from all of the participants and a guaranteed monetary compensation (80 yuan RMB) was given to every subject. During analysis, the outlying data of one participant was excluded, leaving a remaining group of 30 healthy individuals (16 males; mean age ±*SD* = 22.6 ± 1.82). All procedures were approved by the ethics committee of the Institute of Psychology, Chinese Academy of Sciences.

### Materials

We selected 36 negative pictures and 36 neutral pictures from the International Affective Pictures System (IAPS; Lang et al., 2008), in which the normative ratings of valence for each picture range from 1 (negative) to 9 (positive) and ratings of arousal range from 1 (calm) to 9 (arousal). In our study, the selected negative pictures had a mean valence rating of 2.77 (*SD* = 0.67), and a mean arousal rating of 6.49 (*SD* = 0.29). The neutral pictures had a mean valence rating of 5.29 *(SD* = 0.12), and a mean arousal rating of 3.34 (*SD* = 0.39). The pictures following the certain cue and uncertain cue were different. The valence and arousal ratings of the 24 negative pictures recruited in certain-negative trials (valence rating: 2.77 ± 0.69; arousal rating: 6.49 ± 0.27) and those of the 12 negative pictures recruited in uncertain-negative trials (valence rating: 2.78 ± 0.65; arousal rating: 6.49 ± 0.33) were not significantly different [valence rating: *t*(34,1) = -0.06, *p* = 0.95; arousal rating: *t*(34,1) = 0.050, *p* = 0.96]. Also, the valence and arousal ratings of the 24 neutral pictures presented on certain-neutral trials (valence rating: 5.27 ± 0.11; arousal rating: 3.38 ± 0.38) and those of the 12 neutral pictures presented on uncertain-neutral trials (valence rating: 5.34 ± 0.13; arousal rating: 3.27 ± 0.40) were not significantly different [valence rating: *t*(34,1) = -1.56, *p* = 0.13; arousal rating: *t*(34,1) = 0.828, *p* = 0.41]. Differences in the valence and arousal ratings between aversive pictures and neural pictures for certain trials [valence rating: *t*(34,1) = -17.61, *p* < 0.001, *d* = -5.06; arousal rating: *t*(34,1) = 32.86, *p* < 0.001, *d* = -9.43], were similar to corresponding differences for uncertain trials [valence rating: *t*(34,1) = -13.31, *p* < 0.001, *d* = -5.46; arousal rating: *t*(34,1) = 21.26, *p* < 0.001, *d* = -8.78].

### Paradigm

**FIGURE 1:**
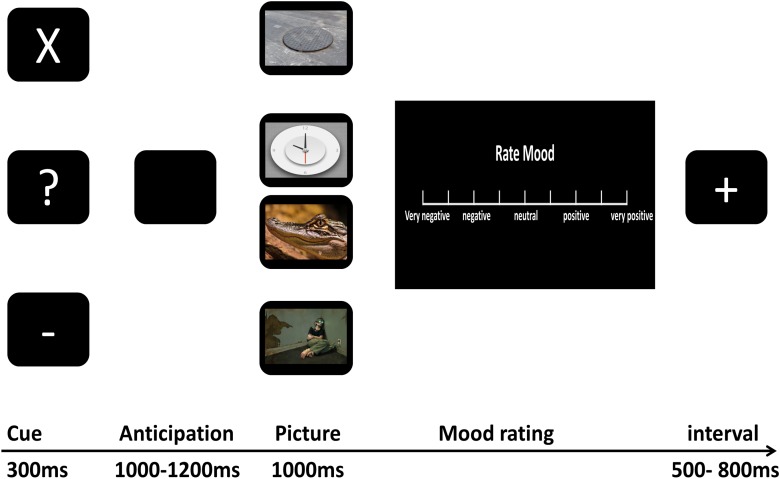
The experimental procedure.

As shown in Figure 1, we used a cue-picture paradigm similar to that used in the study of Sarinopoulos et al. (2010). First, one of three white characters, “X,” “-,” “?” was presented on a black screen for 300 ms indicating the kind of the upcoming picture on each trial. The cross and minus marks were always followed by certain-neutral and certain-negative pictures, respectively, while the question marks were followed by either neutral or negative pictures according to a 50/50 ratio. Participants were explicitly informed about the nature of cue-picture pairings for certain-negative and -neutral pictures, however, they were not told about the precise presentation probability of negative or neutral pictures that followed question marks (uncertain cues). The inter-stimulus interval featured a black screen and ranged from 1000 ms to 1200 ms to evoke participants’ anticipation for the impending stimulus. Then, the picture corresponding to the cue that preceded it was presented on a black screen for 1000 ms. Following picture presentation, participants were immediately asked to rate how the picture affected their mood by turning a dial with their right hand to move a cursor along the VAS. The scale consisted of nine rulings ranging from the first (“very negative”), third (“negative”), intermediate (“neutral”), seventh (“positive”) to the last (“very positive”), scoring “0–100” from the left to the right of the scale continuously. Once participants made a response, the scale was replaced by a jittered 500 ms to 800 ms inter-trial interval featuring a black screen and a white plus sign.

The procedure consisted of three runs, and each run was made up of 24 certain-neutral trials, 24 certain-negative trials, 12 uncertain-negative trials, and 12 uncertain neutral trials. The whole course of the experiment lasted about 40 min. At the end of each run, a scale (from 0 to 100) was presented on the screen with the question “What was the percentage of question marks followed by negative pictures?” This scale was used to compute participants’ post-experiment estimation of uncertain threat. For each kind of trial (certain-negative trial, certain-neutral trial, uncertain-negative trial, and uncertain-neutral trial), the order of the pictures was randomized in each run and reshuffled across the different runs, and each picture was presented three times. Participants conducted the task in a sound-proof and electrically shielded room. Before the formal experiment began, participants performed three practice blocks to familiarize themselves with the paradigm. ERPs were recorded during the whole process.

### EEG Recording

Continuous EEG data was recorded with the Neuroscan Synamp2 Amplifier and 64 Ag/AgCl electrodes in accordance with the 10/20 system. All signals were digitized using a band-pass filter of 0.05–100 Hz and at a sample rating of 1000 Hz in AC mode. All electrodes were on-line referenced to the left mastoid. Electrode impedance was kept below 5 kΩ throughout the experiment. The vertical and horizontal electrooculograms (EOG) were recorded for the offline eye-movement correction of the data. The electrodes on the supra-orbital and infra-orbital places of the left eye were recorded as the vertical EOG, while the electrodes on the outer canthi of both eyes were recorded as the horizontal EOG.

### Data Analysis

We conducted the offline analysis using Neuroscan 4.3. Raw data were off-line re-referenced to half recorded right mastoid, such that the data were finally referenced to an average of the original left and right mastoids through on-line and off-line references. After removing ocular artifacts using the default parameter of the SCAN 4.3 ocular artifact tool, a low pass filter of 16 Hz (24 dB/oct, zero phase filter) was conducted.

Signal data were epoched into cue-locked phase, the duration since the onset of the cue and before the presentation of picture, and picture-locked phase, the duration since the onset of the picture and before the next trial. During cue-locked phase, we analyzed the component SPN. The averaged epoch was 1500 ms with a 200 ms pre-cue onset as the baseline, 300 ms presentation of the cue and 1000 ms after the presentation of cue. Based on previous studies (Wynn et al., 2010; Lin et al., 2014) and visual detection, the following 12 electrodes were selected to conduct statistical analysis: AF3, FPZ, AF4, F3, FZ, F4, FC3, FCZ, FC4, C3, CZ, C4. The mean amplitude of SPN was calculated in time window 800–1300 ms. Three within-subject factors (Cue condition: certain-negative cue, certain-neutral cue, uncertain cue; Site: prefrontal area, frontal area, frontal–central area, central area; Laterality: left line area, midline area, right line area) were recruited to the three-way repeated measures analysis of variance (ANOVA). The factor Cue condition was used to detect the differences among the processing of different conditions, the factor Site was used to examine the brain distribution of the component SPN, and the Laterality was used to check the lateralized effect of the component SPN. During the picture-locked phase, we analyzed components P2 and LPP. The averaged epoch was 1200 ms with a 200 ms pre-stimulus onset as the baseline and 1000 ms presentation of pictures. Based on previous literature (Lin et al., 2012, 2015; Dieterich et al., 2017) and the visual inspection, the two components were measured in the following electrodes: F3, FZ, F4, FC3, FCZ, FC4, C3, CZ, C4 (P2); F3, FZ, F4, FC3, FCZ, FC4, C3, CZ, C4, CP3, CPZ, CP4, P3, PZ, P4 (LPP). For P2, we selected the time window 100–250 ms to calculate the peak amplitude and latency. Data of both peak amplitude and latency were entered into a four-way repeated measures ANOVA. The four within-subject factors were Certainty (certain, uncertain), Valence (negative, neutral), Site (frontal area, frontal–central area, central area), and Laterality (left line, midline, right line) to explore the manipulation effect of Certainty and Valence on P2 and the brain distribution and lateralization of P2. For LPP, we chose the time window 300–700 ms to analyze the mean amplitude. A four-way repeated measures ANOVA was conducted with factors of Certainty (certain, uncertain), Valence (negative, neutral), Site (frontal area, frontal–central area, central area, central–parietal area, parietal area), and Laterality (left line area, midline area, right line area). The Greenhouse-Geisser epsilon was applied to correct all ANOVA results.

For behavioral mood ratings, data were subjected to a two-way repeated measures ANOVA. The two within-subject factors (Certainty: certain, uncertain; Valence: negative, neutral) were implemented to analyze how certainty of condition and valence of pictures modulate the emotional experiences of individuals.

One of our hypotheses was that more negative mood ratings of pictures were related to altered anticipatory activity, so we first averaged the mean amplitudes of SPN for each condition across all selected electrodes. Then, we regressed the grand mean amplitude of SPN elicited by uncertain cues relative to certain-negative cues against mood ratings of uncertain-negative pictures relative to certain-negative pictures. Similarly, we regressed the grand mean amplitude of SPN elicited by uncertain cues relative to certain-neutral cues against mood ratings of uncertain-neutral pictures relative to certain-neutral pictures. To explore the modulated effect of anticipation on the neural processing of pictures, we first averaged the mean amplitude of LPP and the peak amplitude of P2 for each condition across all selected electrodes to get the grand mean amplitude of LPP and the grand peak amplitude of P2. Then, linear regressions were performed, regressing the grand mean amplitude of SPN elicited by uncertain cues relative to certain-negative cues against the grand mean amplitude of LPP and the grand peak amplitude of P2 yielded by uncertain-negative pictures relative to certain-negative pictures, respectively. Similarly, linear regressions were performed between the grand mean amplitude of SPN elicited by uncertain cues compared to certain-neutral cues and the grand mean amplitude of LPP and the grand peak amplitude of P2 yielded by uncertain-neutral pictures relative to certain-neutral pictures, respectively.

To test for the presence of a covariation bias of uncertainty and negative pictures, we tested the post-experimental estimations of participants against a 50% baseline using a one-sample *t*-test, with a higher score than 50% reflecting a bias. Then, we conducted the regression between the grand mean amplitude of SPN produced by uncertain cues and scores of post-experimental estimations. If there was a correlation, we further regressed the grand mean amplitude of SPN produced by certain-negative cues and certain-neutral cues against scores of post-experimental estimations, to explore whether the relationship between the uncertain anticipatory activity and the covariation bias was specific. To explore whether mood contributes to covariation bias, we regressed mood ratings of uncertain-negative pictures relative to certain-negative pictures against post-experimental estimations. Similarly, we regressed mood ratings of uncertain-neutral pictures relative to certain-neutral pictures against post-experimental estimations.

## Results

### Behavioral Data

**FIGURE 2:**
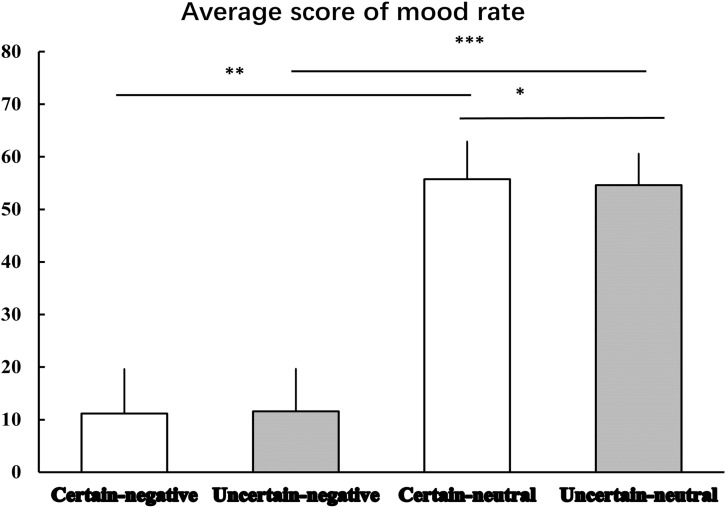
The average scores for mood rating. Error bars represent standard error of mean. ‘^∗^’ indicates the *P*-value of statistical result of uncertain-neutral pictures relative to certain-neutral pictures, *p* = 0.079; ‘^∗∗^’ indicates the *P*-value of statistical result of certain-negative pictures relative to certain-neutral pictures, *p* < 0.001; ‘^∗∗∗^’ indicates the *P*-value of statistical result of uncertain-negative pictures relative to uncertain-neutral pictures, *p* < 0.001.

Figure 2 displays the results of mood rating. The Certainty by Valence repeated measures ANOVA produced a significant main effect of Valence [*F*(1,29) = 564.96, *p* < 0.001, ${\text{η}}_{\text{p}}^{2}$ = 0.95], indicating that mood elicited by negative pictures was more negative than that elicited by neutral pictures. Although, there was no significant main effect of Certainty [*F*(1,29) = 1.01, *p* = 0.324, ${\text{η}}_{\text{p}}^{2}$ = 0.03], we did observe an interaction effect between Certainty and Valence was marginal significant [*F*(1,29) = 3.97, *p* = 0.058, ${\text{η}}_{\text{p}}^{2}$ = 0.12]. Simple analysis showed that the Valence factor was still significant under both certain [*t*(29) = -22.20, *p* < 0.001, *d* = -31.17] and uncertain [*t*(29) = -24.57, *p* < 0.001, *d* = -33.12] conditions. That is, mood ratings of negative pictures were rated more negatively than mood ratings of neutral pictures. The Certainty factor played a significant role in emotional responses to neutral pictures. That is, mood ratings of uncertain-neutral pictures were rated more negatively than mood ratings of certain-neutral pictures [*t*(29) = -1.82, *p* = 0.079, *d* = -0.93]. This uncertainty-related effect was not detected for negative pictures, where we observed a non-significant difference between mood ratings of uncertain-negative pictures and certain-negative pictures [*t*(29) = 1.06, *p* = 0.299, *d* = 0.28].

By comparing post-experimental estimation score against a 50% baseline using a one-sample *t*-test, we observed a covariation bias. Participants estimated the percentage of negative pictures that followed uncertain cues as 65% (*SD* = 17.84), significantly larger than the real probability 50% [*t*(29) = 4.57, *p* < 0.001].

### ERP Data

#### Cue-Locked ERPs

##### SPN

**FIGURE 3:**
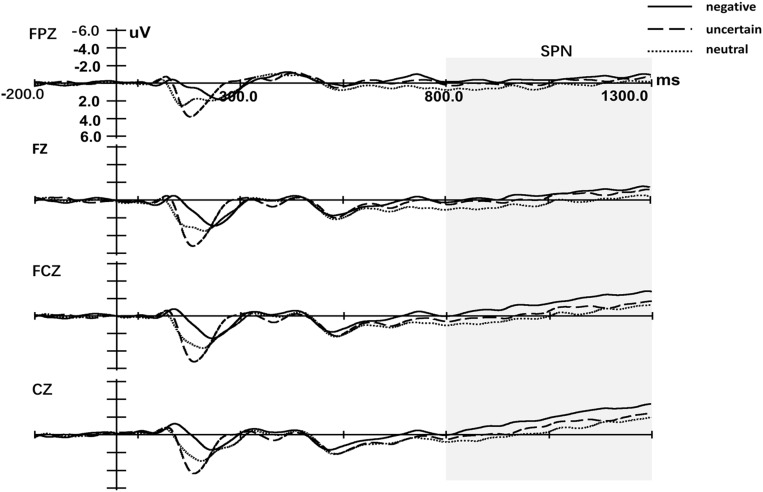
Grand-averaged event-related potentials in midline electrodes during cue-locked phase. Negative represents the certain-negative cue, uncertain represents the uncertain cue and neutral represents the certain-neutral cue.

Figure 3 displays the cue-locked ERP of SPN. Three-way repeated measures ANOVA of SPN exhibited a significant main effect for both the Cue [*F*(1.99,57.84) = 6.01, *p* = 0.004, ${\text{η}}_{\text{p}}^{2}$ = 0.17] and Laterality [*F*(1.27,36.57) = 39.48, *p* < 0.001, ${\text{η}}_{\text{p}}^{2}$ = 0.58]. The main effect for Site was not significant [*F*(1.48,42.74) = 2.46, *p* = 0.112, ${\text{η}}_{\text{p}}^{2}$ = 0.08]. *Post hoc* analyses of Cue indicated that the mean amplitude of SPN for certain-neutral cues was less negative than for both certain-negative cues [*t*(29) = 3.50, *p* = 0.002, *d* = 2.93] and uncertain cues [*t*(29) = 2.12, *p* = 0.043, *d* = 1.77], while the amplitude for uncertain cues was not different from the amplitude for certain-negative cues [*t*(29) = -1.31, *p* = 0.202, *d* = -1.14]. The only significant interaction effect was detected between Site and Laterality [*F*(3.11,90.27) = 5.91, *p* = 0.001, ${\text{η}}_{\text{p}}^{2}$ = 0.17]. Simple analysis of the interaction between Site and Laterality indicated that SPN was right lateralization on every Site (all *p* < 0.034) and was larger at frontal–central and central electrodes than at anterior-frontal and frontal electrodes on central and right hemisphere (all *p* < 0.087).

#### Picture-Locked ERPs

##### P2

**FIGURE 4:**
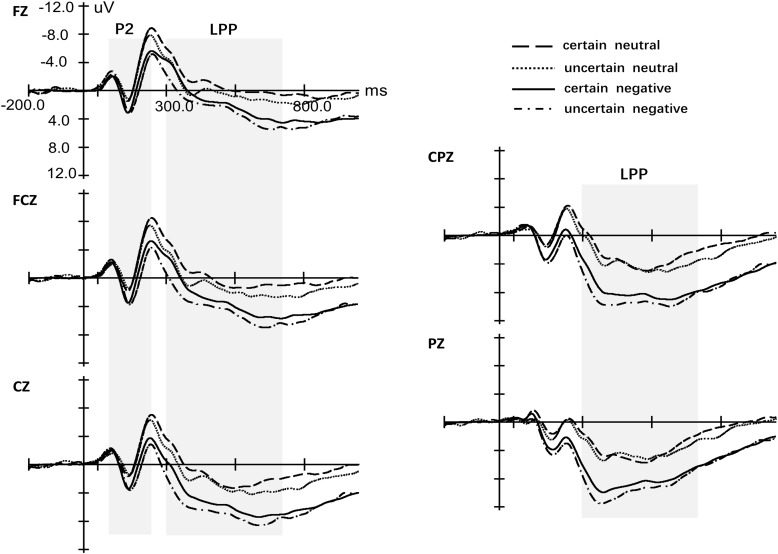
Grand-averaged event-related potentials in midline electrodes during picture-locked phase. The four conditions indicate the four kinds of pictures, certain-neutral picture, uncertain-neutral picture, certain-negative picture and uncertain-negative picture.

ANOVA of the peak for P2 with four factors (Certain, Valence, Site, and Laterality) revealed only a significant main effect of Valence [*F*(1,29) = 23.29, *p* < 0.001, ${\text{η}}_{\text{p}}^{2}$ = 0.46, see Figure 4]. The effects for Certainty [*F*(1,29) = 0.05, *p* = 0.832, ${\text{η}}_{\text{p}}^{2}$ < 0.01], Site [*F*(1.08,31.29) = 2.38, *p* = 0.13, ${\text{η}}_{\text{p}}^{2}$ = 0.08] and Laterality [*F*(1.71,49.72) = 1.94, *p* = 0.160, ${\text{η}}_{\text{p}}^{2}$ = 0.06] were not significant. Also, we did not observe any significant interaction effect. Repeated measures ANOVA of latency for P2 yielded a significant main effect for Certainty [*F*(1,29) = 8.65, *p* = 0.006, ${\text{η}}_{\text{p}}^{2}$ = 0.23], Valence [*F*(1,29) = 13.39, *p* < 0.001, ${\text{η}}_{\text{p}}^{2}$ = 0.32] and Site [*F*(1.15,33.28) = 7.42, *p* = 0.008, ${\text{η}}_{\text{p}}^{2}$ = 0.20] but not Laterality [*F*(1.43,41.33) = 0.95, *p* = 0.370, ${\text{η}}_{\text{p}}^{2}$ = 0.03]. There was a marginal significant interaction between Certainty and Valence [*F*(1,29) = 2.64, *p* = 0.115, ${\text{η}}_{\text{p}}^{2}$ = 0.08]. Simple analysis indicated that uncertainty postponed the latency of P2 for neutral pictures [*t*(29) = 3.37, *p* = 0.002, *d* = 2.39] while there was no difference of latency for P2 between uncertain-negative pictures and certain-negative pictures [*t*(29) = 1.00, *p* = 0.323, *d* = 0.65]. Negative pictures evoked slower P2 than neutral pictures under both certain [*t*(29) = 3.27, *p* = 0.003, *d* = 2.86] and uncertain conditions [*t*(30) = 2.67, *p* = 0.012, *d* = 1.69].

##### LPP

There was a significant main effect of Valence [*F*(1,29) = 56.93, *p* < 0.001, ${\text{η}}_{\text{p}}^{2}$ = 0.67] that negative pictures developed more positive LPP than neutral pictures and Certainty [*F*(1,29) = 8.76, *p* = 0.006, ${\text{η}}_{\text{p}}^{2}$ = 0.23] that uncertainty enlarged the amplitude of LPP for negative and neutral pictures (see Figure 4 and Supplementary Figure S1). However, there was no interaction effect between Valence and Certainty [*F*(1,29) = 2.41, *p* = 0.13, ${\text{η}}_{\text{p}}^{2}$ = 0.08]. The main effect of Laterality [*F*(1.37,39.76) = 0.95, *p* = 0.650, ${\text{η}}_{\text{p}}^{2}$ = 0.02] was not significant while there was a significant main effect of Site [*F*(1.17,33.83) = 56.43, *p* < 0.001, ${\text{η}}_{\text{p}}^{2}$ = 0.66]. *Post hoc* analysis showed that the mean amplitude of LPP increased gradually from frontal area to parietal area (all *p* < 0.017). In addition, Valence significantly interacted with Site [*F*(1.25,36.32) = 20.48, *p* < 0.001, ${\text{η}}_{\text{p}}^{2}$ = 0.41]. Simple analysis indicated that the effect of Valence exerted itself at every level of Site (all *p* < 0.001) while the distributions of LPP were different for negative and neutral pictures. For neutral pictures, LPP increased gradually from frontal area to parietal area and the largest LPP was observed at the parietal region (all *p* < 0.001). For negative pictures, LPP increased gradually from frontal area to central–parietal area and the largest LPP was observed at the central–parietal and parietal regions (all *p* < 0.001) while there was no significant difference between the two regions (*p* = 0.127).

### Regression Data

**FIGURE 5:**
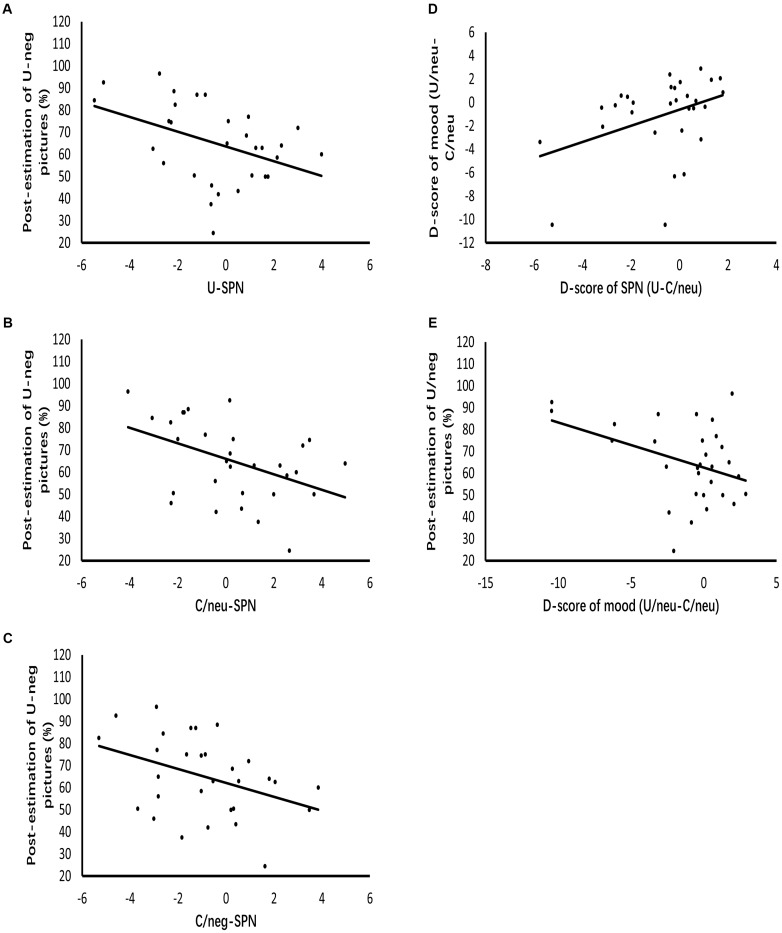
Diagrams of regressions. **(A–C)** Represent the modulation effects of SPN evoked by uncertain cues, certain-neutral cues and certain-negative cues on the post-experimental estimations of negative pictures following uncertain cues, respectively. **(D)** Indicates that enlarged SPN exhibited by uncertain cues relative to certain-neutral cues positively related to the more negative mood ratings of uncertain-neutral pictures relative to certain-neutral pictures. **(E)** Represents that the more negative mood ratings of uncertain-neutral pictures relative to certain-neutral pictures contributes to the post-experimental estimations of negative pictures following uncertain cues.

Figure 5 displays the results of regression analysis. The grand mean amplitude of SPN produced by uncertain cues relative to certain-neutral cues modulated mood ratings of uncertain-neutral pictures relative to certain-neutral pictures (β = 0.388, *p* = 0.034, *R*^2^= 0.12). This result indicates that the larger the difference in anticipatory activities between uncertain and certain-neutral conditions, the more negative the mood ratings were for uncertain-neutral pictures compared to certain-neutral pictures. However, this modulated effect was not detected between the grand mean amplitude of SPN for uncertain cues relative to certain-negative cues and the mood ratings of uncertain-negative pictures relative to certain-negative pictures. There was also no significant result found for the regression analyses between neural anticipatory activities and the neural processing of pictures (including P2 and LPP).

Analysis of the contributors to covariation bias showed that the grand mean amplitude of SPN elicited by uncertain cues positively modulated post-experimental estimations (β = -0.42, *p* = 0.021, *R*^2^= 0.15), indicating that the larger the anticipation for uncertain threat was, the larger the covariation bias became. To detect whether this modulation effect of uncertain anticipation was specific to covariation bias, we further regressed the grand mean amplitude of SPN elicited by certain-negative cues and certain-neutral cues against the covariation bias, respectively. Results showed that covariation bias was also modulated by anticipatory activity for certain-negative pictures (β = -0.39, *p* = 0.033, *R*^2^= 0.12) as well as anticipatory activity for certain-neutral pictures (β = -0.44, *p* = 0.014, *R^2^*= 0.17). Regression analyses of emotion and covariation bias showed that mood ratings of uncertain-neutral pictures compared to certain-neutral pictures contributed to covariation bias (β = -0.39, *p* = 0.034, *R*^2^= 0.12), while mood ratings of uncertain-negative pictures compared to certain-negative pictures was not related to covariation bias.

## Discussion

By recording the temporal dynamics of processing under uncertainty, our study explored two primary questions. First, how is anticipatory activity influenced by uncertainty and what is the subsequent effect of anticipation on neural and behavioral responses to pictures. Second, whether there was a covariation bias of uncertainty and threat and what were the possible contributors to this covariation bias. Self-report data demonstrated a more negative mood rating of uncertain-neutral pictures than certain-neutral pictures, indicating that emotional experiences under the uncertain condition were notably less positive. We also observed a covariation bias such that individuals overestimated the probability of negative pictures following uncertain cues. During the cue stage, we detected that neural activity, specifically in the SPN component, as elicited by certain-negative cues and uncertain cues, was increased relative to that elicited in response to certain-neutral cues. In particular, we found that the amplitudes of SPN evoked by uncertain cues and certain-negative cues were comparable, indicating how anticipatory activity was altered by uncertainty. During the picture stage, larger amplitudes of P2 and LPP were evoked by negative pictures compared to neutral pictures independent of condition. Moreover, uncertainty enlarged the positive deflection of LPP for both negative and neutral pictures. Regression analyses demonstrated that the enlarged SPN elicited by uncertain cues relative to certain-neutral cues positively modulated the more negative mood ratings of uncertain-neutral pictures relative to certain-neutral pictures. More importantly, we observed that the magnitude of the covariation bias was related to general arousal anticipatory activities and the more negative mood ratings of uncertain-neutral pictures compared to certain-neutral pictures. These novel findings give further credibility to the notion that uncertainty can contribute to stress and anxiety-like behaviors.

In the current study, participants reported more negative mood rating in response to negative pictures under both certain and uncertain conditions, a pattern which coincides with the valence of these pictures. However, to our surprise, participants reported similar negative feelings when negative pictures appeared following uncertain cues relative to those following certain cues, a finding which runs counter to that of Grupe and Nitschke (2011). Such a result may have occurred because the pictures we used were not sufficiently unpleasant to produce the previously reported uncertainty-related effect. For example, the aversiveness of stimuli has been found to influence both expectancy and post-experimental estimation (Grupe and Nitschke, 2011; Wiemer et al., 2014). Emotional valence is also known to affect uncertainty-related neural activities (Lin et al., 2015). Although, we selected negative pictures with a significant level of aversiveness compared to the neutral set, those employed by Grupe and Nitschke (2011) were more aversive (valence *M* = 2.32, *SD* = 0.71). Similar with our research, the study of Dieterich et al. (2016), in which the valence of the pictures was less negative (valence *M* = 2.80, *SD* = 0.38) than that of the study of Grupe and Nitschke (2011), also did not produce an uncertainty-related effect, showing no significant difference in mood ratings between certain and uncertain aversive pictures. This is also verified by our post-experimental interviews in which participants did not perceive the negative pictures to be extremely dreadful. The unexpected finding deserves further exploration and also serves to demonstrate an important point about the use of negative pictures as a tool to induce the experience of threat. Unlike the threat of electric shock which serves as a tool for intrinsically high motivational arousal, whether negative pictures can facilitate the detection of activity associated with the impact of uncertainty on affect is highly dependent on both their valence and arousal. In addition, the influence of cultural differences on anxiety (Heinrichs et al., 2006; Hoge et al., 2006) should be considered. That the current experiment was conducted on Chinese students, while that of Grupe and Nitschke (2011) was conducted on multi-racial populations may go some way to explaining the disparity in the effect of uncertainty on mood ratings between these studies.

As hypothesized, we detected a covariation bias whereby participants overestimate the proportion of negative pictures that followed uncertain cues (represented as question marks). Consistent with some previous work (Dieterich et al., 2016), even though participants were exposed to the relationship of uncertain cues and negative pictures on multiple occasions, they were unable to objectively learn the contingency of forthcoming threat. This covariation bias is fairly important, particularly considering that it might passively contribute to participants’ psychological and behavioral activity when facing future uncertain events and is shown to be related to state anxiety (Wiemer et al., 2014).

During the cue phase, we found that anticipation for certain-negative pictures and uncertain-negative pictures elicited larger SPN compared to anticipation for certain-neutral pictures. In particular, the anticipation for uncertain-negative pictures reflected by SPN was not different from anticipation for certain-negative pictures, though the occurrence of negative pictures under uncertainty was 50%. The mean amplitude of SPN is related to the attention resources required to prepare and anticipate upcoming events and varies according to the affective content of the subsequent stimulus (Bradley et al., 2001), with larger elicited by emotionally salient stimuli (Simons et al., 1979; Klorman and Ryan, 1980; Lumsden et al., 1986; Amrhein and Pauli, 2005). This was also demonstrated in our study which showed that anticipation for negative pictures elicited larger SPN than that for neutral pictures. However, when the occurrence of negative pictures was uncertain, participants’ anticipatory activity was similar to that for certain-negative pictures. Such a pattern of results may indicate an expectancy bias for negative pictures under uncertainty from participants.

During the early processing of pictures, we found that negative pictures evoked a larger amplitude of P2 than neutral pictures, regardless of whether they occurred after certain or uncertain cues. This is consistent with previous findings suggesting that P2 was enlarged by negative emotional stimuli (Delplanque et al., 2004; Huang and Luo, 2006; Dan and Hajcak, 2008; Carretie et al., 2013). However, unlike the study of Dieterich et al. (2016) who found that uncertain-negative pictures produced larger P2 than certain-negative pictures, we did not detect an uncertainty-related effect on the amplitude of P2. Our result was, however, consistent with other work from Dieterich et al. (2017), in which the uncertainty-related effect was only observed in obsessive-compulsive disorder (OCD) but not in healthy participants, suggesting that the uncertainty-related effect on early attention to threat is not stable in healthy participants. Future studies should explore this problem by comparing effects between healthy participants and anxiety patients. Another interesting finding of P2 was that negativity and uncertainty delayed its latency. Specifically, the latency for negative pictures was larger relative to neutral pictures under both certain and uncertain conditions. The latency of P2 for uncertain-neutral pictures was also larger relative to certain-neutral pictures while this was not the case for uncertain-negative pictures relative to certain-negative pictures. The peak latency of P2, as an indicator of the time required for the perceptual analysis at the early stage of processing, is slower when the processing of visual information is less efficient (Yuan et al., 2011; Yang et al., 2012). Thus, we cautiously propose that the observations of reduced efficiency in perceiving both negative pictures relative to neutral pictures and uncertain-neutral pictures relative to certain-neutral pictures might be a demonstration of the ‘avoidance motive’ as caused by the belief of the occurrence of a negative outcome. For example, when participants were certain about the negative valence of forthcoming pictures under the certain-negative condition, in their mind, they were unwilling to process them, thus when the picture actually appeared, they postponed their responses. On the other hand, when the condition was uncertain, participants may have been inclined to hold a “pessimistic” opinion given the high probability of a negative outcome (Grupe and Nitschke, 2011). As a result, the processing of uncertain-neutral pictures was put off relative to certain-neutral pictures. The uncertainty-related effect was detected at the late stage of processing as reflected by the larger amplitude of LPP under the uncertain condition relative to the certain condition. The uncertainty-related effect for neutral pictures was also detected, thus verifying the study of Dieterich et al. (2016, 2017) that uncertainty enhances attention allocation to stimuli.

Importantly, by examining the relationship between anticipatory activity leading up to pictures and the subsequent emotional responses to these pictures, we found that the mean amplitude of SPN evoked by uncertain cues relative to certain-neutral cues was positively related to the mood ratings of uncertain-neutral pictures relative to certain-neutral pictures. This indicates that the magnification of anticipatory activity under uncertainty contributes to the increased negative characteristics of emotional experience. As demonstrated by previous studies, larger SPN is related to the emotional salience of forthcoming stimuli (Simons et al., 1979; Klorman and Ryan, 1980; Lumsden et al., 1986; Amrhein and Pauli, 2005). This was also verified in our findings whereby, under the certain condition, a larger SPN was found prior to negative pictures relative to neutral pictures. Thus, the enlarged anticipation under uncertain condition relative to certain neutral condition (as indicated by a larger amplitude of SPN) might be a result of or at least partly influenced by the expectancy of an emotionally salient picture, which further contributes to the more negative emotional experience after viewing the subsequent neutral pictures (as indicated by more negative mood ratings). However, this modulation effect was not detected for negative pictures under uncertain condition relative to certain condition. One explanation may be linked to the less aversive nature of the negative pictures included in our study, as described above. This may lead to a failure in detecting the uncertainty-related effect on mood ratings of negative pictures as well as a failure in detecting the subsequent modulation effect. Alternatively, the anticipatory activity under uncertainty might be more closely related to the occurrence of negative pictures without their associated costs. That is, prior to the experiment, we instructed participants that when there was a “?” mark, the subsequent picture might be either negative or neutral. Thus, when participants saw the “?” mark during the experiment, their first and automatic reaction might be the anticipation of the occurrence of a negative picture. If the subsequent actual outcome was a neutral picture, rather than simply processing the picture as neutral as would be the case under the certain condition, participants may, to some degree at least, process the picture as negative. Participants may subsequently report more negative emotional experiences in response to neutral picture under uncertain condition relative to certain condition. However, when the actual outcome was a negative one as predicted, they just processed it as they would process the negative pictures under certain condition.

Surprisingly, and contrary to previous work (Sarinopoulos et al., 2010), we did not observe any relationship between anticipatory activities and the neural processing of pictures. The study of Sarinopoulos et al. (2010) found the anticipatory activity of ACC activated by uncertain cues relative to certain-negative cues was inversely related to activities of insula and amygdala activated by uncertain-negative pictures relative to certain-negative pictures, indicating a regulatory role of anticipation on subsequent processing of pictures. However, this regulatory effect of anticipation (reflected by SPN) on neural processing of pictures (reflected by P2 and LPP) was not detected in our study. Distinct neural pathways have been demonstrated during the anticipation of pain. Using fMRI, the study of Ploghaus et al. (2003) showed that, when the occurrence of a shock was certain, expectation was associated with activities in the posterior cerebellum and rostral ACC, while, when the occurrence of a shock was uncertain, expectation was associated with activities in the mid-cingulate cortex, ventromedial prefrontal cortex and hippocampus. Brown et al. (2008) used ERP to explore the anticipation of uncertain pain, and the source analysis suggested that uncertain anticipation engaged cortical areas implicated in attention, while certain anticipation appeared to engage cortical areas more closely related to semantic and prospective memory. This separation might be the reason why we could not detect the relationship between anticipatory activity and neural responses to pictures. Further studies are needed to explore this question.

The findings of altered anticipation under uncertainty at the neural level and its modulation effect on emotional experience highlight the important role of anticipation and the need to pay more attention to associated neural activity. One common feature shared by individuals with anxiety disorders is the presence of pessimistic beliefs about future negative events (Nitschke et al., 2009). This kind of anticipation bias has also been detected during anticipation for uncertain threat in healthy participants (Grupe and Nitschke, 2011; Dieterich et al., 2016). Taken together, these offer a novel insight into the understanding of how uncertainty might contribute to the pathological basis of anxiety disorders. For example, anxious individuals usually have irrational cognition of uncertain threat and appraise stimuli through a “stretched” dimension, such that they rate fear-relevant stimulus as more threatening, fear-irrelevant stimulus as safer and intermediate threat stimulus as more threatening than non-anxious individuals do (Halberstadt and Niedenthal, 1997; Cavanagh and Davey, 2001; Davey, 2006). In our study, more negative mood ratings were reported after uncertain-neutral pictures relative to certain-neutral pictures and more attention resources were allocated to uncertain stimuli relative to certain stimuli (as reflected by the enlarged mean amplitude of LPP), which might indicate the increased emotional intensity under uncertainty (Schupp et al., 2010). Such a pattern may have parallels with the “stretched” emotional dimension utilized by anxious individuals. Since we have only currently explored the affective responses to uncertain threat in healthy individuals, future studies could benefit from probing these problems in anxious individuals. This would allow the identification of key differences between groups in the processing of uncertain threat at neural and behavioral level. Further, though the uncertain anticipation bias was reported in previous studies (Grupe and Nitschke, 2011; Dieterich et al., 2016) and the altered neural anticipatory activity under uncertainty was demonstrated in our study, the direct evidence of the relationship between negative belief and neural anticipatory activity is still lack. Researches focusing on this point would allow the further understanding of the role of negative belief in the processing of affection, which will further promote the comprehension of the pathological mechanism of anxiety.

Further, we found that the covariation bias was manipulated by the anticipation of uncertain threat, such that the more participants thought the forthcoming picture would be negative, the more they thought that the uncertain cue was related to the negative picture. This effect occurred even after participants had been exposed to the real relationship between the uncertain cue and the negative result on multiple occasions. However, this modulation effect was not specific. Anticipatory activities elicited by certain-negative and certain-neutral cues also contributed to the covariation bias in the same pattern. Thus, it seems that it is the general arousal activities during anticipation rather than the expectancy bias during uncertain anticipation modulated the covariation bias. Future studies would benefit from exploring the relationship among personal characteristics, anticipation and covariation bias. For example, neuroticism and anxiety sensitivity have been demonstrated to be related to the anticipatory activity (Conrod, 2006; Drabant et al., 2011), but it is still unclear whether these factors lead to general increased anticipatory activities and then contribute to the covariation bias. Another way to verify whether uncertain anticipation is specific to covariation bias would be to exclude the potential influence of personal characteristics.

Importantly, this covariation bias was partly accounted for by a more negative emotional experience. Specifically, participants showing increased negative mood ratings of uncertain-neutral pictures compared to certain-neutral pictures were more likely to give a higher estimation that the uncertain condition was associated with negative pictures. This result demonstrates the relationship between emotion and covariation bias when the situation was uncertain. Thus, the estimation of the contingency between uncertainty and threat may not be simply related to anticipatory activities for outcomes, but could also be influenced by the emotional experience of these outcomes relevant to the current condition. However, we did not detect a relationship between covariation bias and mood ratings of negative pictures under the uncertain condition relative to the certain condition. The absence of such a relationship might be attributed to the non-significant difference in emotional experience between the uncertain-negative pictures and certain-negative pictures, as described above. Future studies might further explore this question by utilizing more salient stimuli, such as an electric shock.

Covariation bias takes on great importance in light of its consistent presence in anxiety disorders (de Jong et al., 1992, 1995a,b, Pauli et al., 1998; Hermann et al., 2004; Amrhein and Pauli, 2005). The study of de Jong et al. (1995a) found that the increased likelihood of relapse in phobic women after treatment was related to their heightened covariation bias of the relationship between fear-relevant stimuli and aversive outcomes. Thus, extensive work could explore whether the covariation bias under uncertainty is a common feature across anxiety disorders and whether there is any difference of the covariation bias between healthy populations and anxiety disorders. This is especially informative considering the possible correlation between covariation bias and the symptomology and treatment of anxiety. Further, the presence of a covariation bias associating uncertainty with negative events might provide a new way of screening and intervening of populations at high risk of anxiety and anxiety disorders. Of particular importance, our findings, by demonstrating the correlation between covariation bias and both the general anticipatory activities and emotional experience, offer possible methods to promote objective contingency awareness and eventually contribute to the prevention or treatment of anxiety.

A few limitations of the current study should be addressed. Firstly, though our instructions about the three kinds of cues were related to the occurrence of negative and neutral pictures, participants’ anticipation for uncertain threat may have been related to both the possibility and cost of future negative events. As a consequence, we were not able to definitively clarify whether the overestimation of the cost of uncertain threat or the high evaluation of the frequency of uncertain threat contributes more to the more negative emotional experience and the associated covariation bias. In order to explore which is more strongly related to the development and maintenance of anxiety, future studies might include stimuli presented under different probabilities of uncertainty and those vary in both valence and arousal. Secondly, all of our participants were young undergraduate and graduate students, thus limiting the generalization of our findings. Further research might benefit from the inclusion of groups belonging to a range of ages and levels of education. Finally, in the current task, the pictures recruited in the uncertain and certain conditions were different. Although we controlled the valence and arousal of pictures following the certain and uncertain trails, methods of counterbalancing pictures across all conditions would be a better way to exclude the possible influence of different pictures.

To summarize, in the present study we observed altered anticipatory activity and covariation bias of individuals under uncertainty and their neural and behavioral responses to potential threat. Importantly, we found that altered anticipation by uncertainty-modulated the subsequent emotional experience and both the general arousal during anticipation of forthcoming stimuli and the emotional experience of these stimuli contributed to covariation bias of uncertainty and threat. These findings highlight the important role of anticipation and covariation bias in exploring how uncertainty might contribute to subclinical and clinical anxiety disorders.

## Author Contributions

ZQ and HG contributed to the conception and design of the study. ZQ, HG, and YW organized the database. ZQ performed the statistical analysis. ZQ wrote the first draft of the manuscript. All authors contributed to manuscript revision, read and approved the submitted version.

## Conflict of Interest Statement

The authors declare that the research was conducted in the absence of any commercial or financial relationships that could be construed as a potential conflict of interest.

## Supplementary Material

FIGURE S1 Topographic maps of voltage differences of LPP in the 300- to 700-ms time ranges. The left one is the voltage difference of LPP between uncertain-negative pictures and certain-negative pictures. The right one is the voltage difference of LPP between uncertain-neutral pictures and certain-neutral pictures.

## References

[B1] Alloy L. B. Tabachnik N. (1984). Assessment of covariation by humans and animals: the joint influence of prior expectations and current situational information. *Psychol. Rev.* 91 112–149. 10.1037/0033-295X.91.1.112 6571422

[B2] Amin J. M. Lovibond P. F. (1997). Dissociations between covariation bias and expectancy bias for fear-relevant stimuli. *Cogn. Emot.* 11 273–289. 10.1080/026999397379926

[B3] Amrhein C. Pauli P. (2005). Covariation bias and its physiological correlates in panic disorder patients. *J. Anxiety Disord.* 19 177–191. 10.1016/j.janxdis.2004.01.004 15533703

[B4] Bar-Anan Y. Wilson T. D. Gilbert D. T. (2009). The feeling of uncertainty intensifies affective reactions. *Emotion* 9 123–127. 10.1037/a0014607 19186925

[B5] Barlow D. H. (1988). Anxiety and its disorders: the nature and treatment of anxiety and panic (2nd ed.). *Clin. Psychol. Rev.* 26 105–106.

[B6] Bradley M. M. Codispoti M. Cuthbert B. N. Lang P. J. (2001). Emotion and motivation I: defensive and appetitive reactions in picture processing. *Emotion* 1 276–298. 10.1037/1528-3542.1.3.276 12934687

[B7] Brown C. A. Seymour B. Boyle Y. El-Deredy W. Jones A. K. (2008). Modulation of pain ratings by expectation and uncertainty: behavioral characteristics and anticipatory neural correlates. *Pain* 135 240–250. 10.1016/j.pain.2007.05.022 17614199

[B8] Buhr K. Dugas M. J. (2002). The intolerance of uncertainty scale: psychometric properties of the English version. *Behav. Res. Ther.* 40 931–945. 10.1016/j.jbtep.2012.07.004 12186356

[B9] Carretié L. Kessel D. Carboni A. Lópezmartín S. Albert J. Tapia M. (2013). Exogenous attention to facial vs non-facial emotional visual stimuli. *Soc. Cogn. Affect. Neurosci.* 8 764–773. 10.1093/scan/nss068 22689218 PMC3791067

[B10] Cavanagh K. Davey G. C. L. (2001). The use of stimulus dimensions in judgement making in spider fearful and nonfearful individuals. *Behav. Res. Ther.* 39 1199–1211. 10.1016/S0005-7967(00)00094-2 11579989

[B11] Conrod P. J. (2006). The role of anxiety sensitivity in subjective and physiological responses to social and physical stressors. *Cogn. Behav. Ther.* 35 216–225. 10.1080/16506070600898587 17189239

[B12] Craig A. D. (2002). How do you feel? interoception: the sense of the physiological condition of the body. *Nat. Rev. Neurosci.* 3 655–666. 10.1038/nrn894 12154366

[B13] Critchley H. D. (2004). The human cortex responds to an interoceptive challenge. *Proc. Natl. Acad. Sci. U.S.A.* 101 6333–6334. 10.1073/pnas.0401510101 15096592 PMC404044

[B14] Critchley H. D. (2005). Neural mechanisms of autonomic, affective, and cognitive integration. *J. Comp. Neurol.* 493 154–166. 10.1002/cne.20749 16254997

[B15] Cuthbert B. N. Schupp H. T. Bradley M. M. Birbaumer N. Lang P. J. (2000). Brain potentials in affective picture processing: covariation with autonomic arousal and affective report. *Biol. Psychol.* 52 95–111. 10.1016/S0301-0511(99)00044-7 10699350

[B16] Dan F. Hajcak G. (2008). Deconstructing reappraisal: descriptions preceding arousing pictures modulate the subsequent neural response. *J. Cogn. Neurosci.* 20 977–988. 10.1162/jocn.2008.20066 18211235

[B17] Davey G. C. L. (2006). “Cognitive mechanisms in fear acquisition and maintenance,” in *Fear and Learning: From Basic Processes to Clinical Implications*, eds Craske M. G. Hermans D. Vansteenwegen D. (Washington, DC: American Psychological Association), 99–116. 10.1037/11474-005

[B18] de Jong P. J. Ma V. D. H. Merckelbach H. (1995a). Covariation bias and the return of fear. *Behav. Res. Ther.* 33 211–213. 7887881 10.1016/0005-7967(94)e0024-d

[B19] de Jong P. J. Merckelbach H. Arntz A. (1995b). Covariation bias in phobic women: the relationship between a priori expectancy, on-line expectancy, autonomic responding, and a posteriori contingency judgment. *J. Abnorm. Psychol.* 104 55–62. 7897053 10.1037//0021-843x.104.1.55

[B20] de Jong P. J. Merckelbach H. Arntz A. Nijman H. (1992). Covariation detection in treated and untreated spider phobics. *J. Abnorm. Psychol.* 101 724–727. 10.1037/0021-843X.101.4.724 1430613

[B21] Delplanque S. Lavoie M. E. Hot P. Silvert L. Sequeira H. (2004). Modulation of cognitive processing by emotional valence studied through event-related potentials in humans. *Neurosci. Lett.* 356 1–4. 10.1016/j.neulet.2003.10.014 14746887

[B22] Desteno D. Petty R. E. Wegener D. T. Rucker D. D. (2000). Beyond valence in the perception of likelihood: the role of emotion specificity. *J. Pers. Soc. Psychol.* 78 397–416. 10.1037/0022-3514.78.3.397 10743870

[B23] Dieterich R. Endrass T. Kathmann N. (2016). Uncertainty is associated with increased selective attention and sustained stimulus processing. *Cogn. Affect. Behav. Neurosci.* 16 447–456. 10.3758/s13415-016-0405-8 26810702

[B24] Dieterich R. Endrass T. Kathmann N. (2017). Uncertainty increases neural indices of attention in obsessive-compulsive disorder. *Depress. Anxiety* 34 1018–1028. 10.1002/da.22655 28543920

[B25] Drabant E. M. Kuo J. R. Ramel W. Blechert J. Edge M. D. Cooper J. R. (2011). Experiential, autonomic, and neural responses during threat anticipation vary as a function of threat intensity and neuroticism. *Neuroimage* 55 401–410. 10.1016/j.neuroimage.2010.11.040 21093595 PMC3031673

[B26] Egner T. Etkin A. Gale S. Hirsch J. (2008). Dissociable neural systems resolve conflict from emotional versus nonemotional distracters. *Cereb. Cortex* 18 1475–1484. 10.1093/cercor/bhm179 17940084

[B27] Etkin A. Egner T. Peraza D. M. Kandel E. R. Hirsch J. (2006). Resolving emotional conflict: a role for the rostral anterior cingulate cortex in modulating activity in the amygdala. *Neuron* 51 871–882. 10.1016/j.neuron.2006.07.029 16982430

[B28] Grupe D. W. Nitschke J. B. (2011). Uncertainty is associated with biased expectancies and heightened responses to aversion. *Emotion* 11 413–424. 10.1037/a0022583 21500909 PMC3086262

[B29] Grupe D. W. Nitschke J. B. (2013). Uncertainty and anticipation in anxiety: an integrated neurobiological and psychological perspective. *Nat. Rev. Neurosci.* 14 488–501. 10.1038/nrn3524 23783199 PMC4276319

[B30] Halberstadt J. B. Niedenthal P. M. (1997). Emotional state and the use of stimulus dimensions in judgment. *J. Pers. Soc. Psychol.* 72 1017–1033. 10.1037/0022-3514.72.5.1017 9150582

[B31] Heinrichs N. Rapee R. M. Alden L. A. Bögels S. Hofmann S. G. Oh K. J. (2006). Cultural differences in perceived social norms and social anxiety. *Behav. Res. Ther.* 44 1187–1197. 10.1002/da.20759 16325145

[B32] Hermann C. Ofer J. Flor H. (2004). Covariation bias for ambiguous social stimuli in generalized social phobia. *J. Abnorm. Psychol.* 113 646–653. 10.1037/0021-843X.113.4.646 15535796

[B33] Hoge E. A. Tamrakar S. M. Christian K. M. Mahara N. Nepal M. K. Pollack M. H. (2006). Cross-cultural differences in somatic presentation in patients with generalized anxiety disorder. *J. Nerv. Ment. Dis.* 194 962–966. 10.1097/01.nmd.0000243813.59385.75 17164637

[B34] Huang Y. X. Luo Y. J. (2006). Temporal course of emotional negativity bias: an ERP study. *Neurosci. Lett.* 398 91–96. 10.1016/j.neulet.2005.12.074 16446031

[B35] Kennedy S. J. Rapee R. M. Mazurski E. J. (1997). Covariation bias for phylogenetic versus ontogenetic fear-relevant stimuli. *Behav. Res. Ther.* 35 415–422. 10.1016/S0005-7967(96)00128-3 9149450

[B36] Klorman R. Ryan R. M. (1980). Heart rate, contingent negative variation, and evoked potentials during anticipation of affective stimulation. *Psychophysiology* 17 513–523. 10.1111/j.1469-8986.1980.tb02290.x 7443917

[B37] Lang P. J. Bradley M. M. Cuthbert B. N. (2008). *International Affective Picture System (IAPS): Affective Ratings of Pictures and Instruction Manual*. Technical Report. No. A-8. Gainesville, FL: University of Florida.

[B38] Lin H. Gao H. Ye Z. Wang P. Tao L. Ke X. (2012). Expectation enhances event-related responses to affective stimuli. *Neurosci. Lett.* 522 123–127. 10.1016/j.neulet.2012.06.022 22710007

[B39] Lin H. Gao H. You J. Liang J. Ma J. Yang N. (2014). Larger N2 and smaller early contingent negative variation during the processing of uncertainty about future emotional events. *Int. J. Psychophysiol.* 94 292–297. 10.1016/j.ijpsycho.2014.10.004 25312204

[B40] Lin H. Jin H. Liang J. Yin R. Liu T. Wang Y. (2015). Effects of uncertainty on ERPs to emotional pictures depend on emotional valence. *Front. Psychol.* 6:1927. 10.3389/fpsyg.2015.01927 26733916 PMC4686648

[B41] Lumsden J. Howard R. C. Fenton G. W. (1986). The contingent negative variation (CNV) to fear-related stimuli in acquisition and extinction. *Int. J. Psychophysiol.* 3 253–261. 10.1016/0167-8760(86)90034-6 3700186

[B42] Mcnally R. J. Heatherton T. F. (1993). Are covariation biases attributable to a priori expectancy biases? *Behav. Res. Ther.* 31 653–658. 10.1016/0005-7967(93)90118-E 8216167

[B43] Nitschke J. B. Dixon G. E. Sarinopoulos I. Short S. J. Cohen J. D. Smith E. E. (2006). Altering expectancy dampens neural response to aversive taste in primary taste cortex. *Nat. Neurosci.* 9 435–442. 10.1038/nn1645 16462735

[B44] Nitschke J. B. Sarinopoulos I. Oathes D. J. Johnstone T. Whalen P. J. Davidson R. J. (2009). Anticipatory activation in the amygdala and anterior cingulate in generalized anxiety disorder and prediction of treatment response. *Am. J. Psychiatry* 166 302–310. 10.1176/appi.ajp.2008.07101682 19122007 PMC2804441

[B45] Pauli P. Wiedemann G. Montoya P. (1998). Covariation bias in flight phobics. *J. Anxiety Disord.* 12 555–565. 10.1016/S0887-6185(98)00033-4 9879035

[B46] Petrovic P. Dietrich T. Fransson P. Andersson J. Carlsson K. Ingvar M. (2005). Placebo in emotional processing— induced expectations of anxiety relief activate a generalized modulatory network. *Neuron* 46 957–969. 10.1016/j.neuron.2005.05.023 15953423

[B47] Phan K. L. Fitzgerald D. A. Nathan P. J. Moore G. J. Uhde T. W. Tancer M. E. (2005). Neural substrates for voluntary suppression of negative affect: a functional magnetic resonance imaging study. *Biol. Psychiatry* 57 210–219. 10.1016/j.biopsych.2004.10.030 15691521

[B48] Ploghaus A. Becerra L. Borras C. Borsook D. (2003). Neural circuitry underlying pain modulation: expectation, hypnosis, placebo. *Trends Cogn. Sci.* 7 197–200. 10.1016/S1364-6613(03)00061-5 12757820

[B49] Poli S. Sarlo M. Bortoletto M. Buodo G. Palomba D. (2007). Stimulus-preceding negativity and heart rate changes in anticipation of affective pictures. *Int. J. Psychophysiol.* 65 32–39. 10.1016/j.ijpsycho.2007.02.008 17395326

[B50] Sarinopoulos I. Dixon G. E. Short S. J. Davidson R. J. Nitschke J. B. (2006). Brain mechanisms of expectation associated with insula and amygdala response to aversive taste: implications for placebo. *Brain Behav. Immun.* 20 120–132. 10.1016/j.bbi.2005.11.006 16472720

[B51] Sarinopoulos I. Grupe D. W. Mackiewicz K. L. Herrington J. D. Lor M. Steege E. E. (2010). Uncertainty during anticipation modulates neural responses to aversion in human insula and amygdala. *Cereb. Cortex* 20 929–940. 10.1093/cercor/bhp155 19679543 PMC2837092

[B52] Schupp H. T. Cuthbert B. N. Bradley M. M. Cacioppo J. T. Ito T. Lang P. J. (2010). Affective picture processing: the late positive potential is modulated by motivational relevance. *Psychophysiology* 37 257–261. 10.1111/1469-8986.3720257 10731776

[B53] Simons R. F. Ohman A. Lang P. J. (1979). Anticipation and response set: cortical, cardiac, and electrodermal correlates. *Psychophysiology* 16 222–233. 10.1111/j.1469-8986.1979.tb02982.x 441216

[B54] Tomarken A. J. Mineka S. Cook M. (1989). Fear-relevant selective associations and covariation bias. *J. Abnorm. Psychol.* 98 381–394. 10.1037/0021-843X.98.4.381 2592672

[B55] Tomarken A. J. Sutton S. K. Mineka S. (1995). Fear-relevant illusory correlations: what types of associations promote judgmental bias? *J. Abnorm. Psychol.* 104 312–326. 10.1037/0021-843X.104.2.312 7790633

[B56] Van Boxtel G. J. M. Böcker K. B. E. (2004). Cortical measures of anticipation. *J. Psychophysiol.* 18 61–76. 10.1027/0269-8803.18.23.61

[B57] Vanoyen W. C. Vrana S. R. (2000). Emotional imagery, the visual startle, and covariation bias: an affective matching account. *Biol. Psychol.* 52 187–204. 10.1016/S0301-0511(00)00027-2 10725563

[B58] Wager T. D. Rilling J. K. Smith E. E. Sokolik A. Casey K. L. Davidson R. J. (2004). Placebo-induced changes in FMRI in the anticipation and experience of pain. *Science* 303 1162–1167. 10.1126/science.1093065 14976306

[B59] Wiemer J. Muhlberger A. Pauli P. (2014). Illusory correlations between neutral and aversive stimuli can be induced by outcome aversiveness. *Cogn. Emot.* 28 193–207. 10.1080/02699931.2013.809699 23829308

[B60] Wynn J. K. Horan W. P. Kring A. M. Simons R. F. Green M. F. (2010). Impaired anticipatory event-related potentials in schizophrenia. *Int. J. Psychophysiol.* 77 141–149. 10.1016/j.ijpsycho.2010.05.009 20573584 PMC2907238

[B61] Yang J. Yuan J. Li H. (2012). Expectation decreases brain susceptibility to fearful stimuli: ERP evidence from a modified emotion evaluation task. *Neurosci. Lett.* 514 198–203. 10.1016/j.neulet.2012.02.094 22426474

[B62] Yuan J. Xu S. Yang J. Liu Q. Chen A. Zhu L. (2011). Pleasant mood intensifies brain processing of cognitive control: ERP correlates. *Biol. Psychol.* 87 17–24. 10.1016/j.biopsycho.2011.01.004 21315134

