# Supplementary figures and images for: Anticipation of Uncertain Threat Modulates Subsequent Affective Responses and Covariation Bias
Source: Front Psychol. 2018 Dec 11;9:2547. doi: 10.3389/fpsyg.2018.02547 (PMC6297831; doi:10.3389/fpsyg.2018.02547)

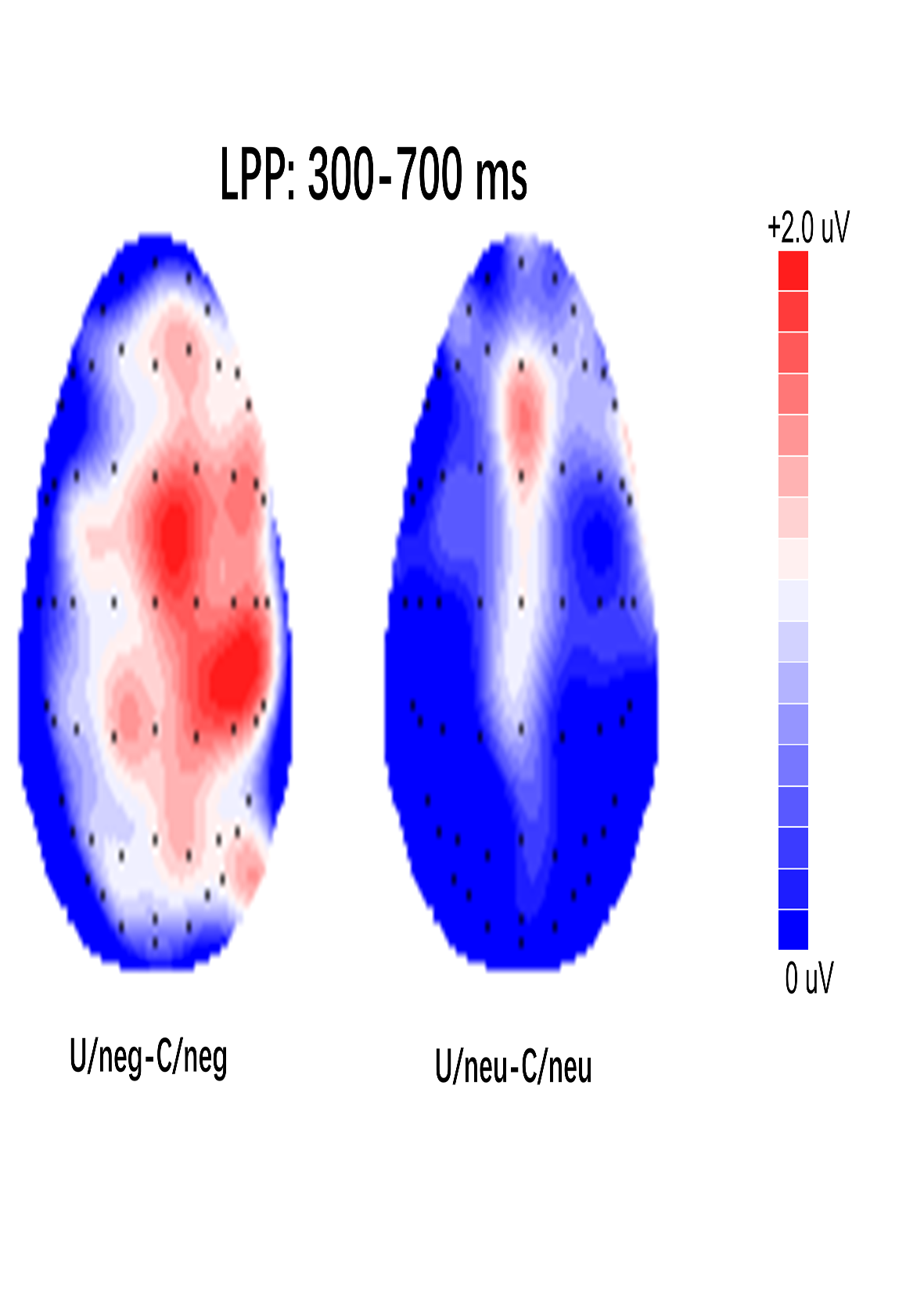

Supplement: FIGURE S1 — Topographic maps of voltage differences of LPP in the 300- to 700-ms time ranges. The left one is the voltage difference of LPP between uncertain-negative pictures and certain-negative pictures. The right one is the voltage difference of LPP between uncertain-neutral pictures and certain-neutral pictures. [file Image_1.TIF]
